# Upper Leg Muscular Co-Contraction During Maximal-Speed Sprinting in Male Club Ice Hockey Athletes

**DOI:** 10.1186/s40798-024-00795-5

**Published:** 2025-01-09

**Authors:** Jason J. Williams, William C. Roshinski, Joseph C. Watso

**Affiliations:** 1 Department of Health, Nutrition, and Food Sciences, Florida State University, Tallahassee, FL USA; 2 Institute of Sports Sciences and Medicine, Florida State University, Tallahassee, FL USA; 3 Cardiovascular & Applied Physiology Laboratory, Department of Health, Nutrition, and Food Sciences, Florida State University, Tallahassee, FL USA; 4 120 Convocation Way Tallahassee, Florida, 32306 USA

**Keywords:** Electromyography, Performance, Muscular co-contraction

## Abstract

**Background:**

Little is known about the lower extremity muscle co-contraction patterns during sprinting and its relation to running velocity (i.e., performance). Therefore, we compared lower extremity muscular activation patterns during sprinting between slower and faster collegiate club hockey athletes. We hypothesized that faster athletes would have lower EMG-assessed co-contraction index (CCI) values in the lower extremities during over-ground sprinting.

**Methods:**

Twenty-two males (age = 21 [1] yrs (median[IQR]); body mass = 77.1 ± 8.6 kg (mean ± SD)) completed two 20-m over-ground sprints with concomitant EMG and asynchronous force plate testing over four days in Tallahassee, Florida, USA. We split participants using median running velocity (FAST: 8.5 ± 0.3 vs. SLOW: 7.7 ± 0.3 m/s, *p* < 0.001).

**Results:**

Faster athletes had lower CCI between the rectus femoris and biceps femoris (group: *p* = 0.05), particularly during the late swing phase of the gait cycle (post hoc *p* = 0.02). Early swing phase duration was moderately inversely related to Hip CCI in the stance phase (ρ=-0.58, *p* < 0.01) and weakly related to Knee CCI in the swing phase (ρ = 0.44, *p* = 0.046). Finally, swing phase duration was moderately inversely related to Hip CCI in the stance phase (ρ=-0.50, *p* = 0.02).

**Conclusions:**

In agreement with our hypothesis, we found lower CCI values in the upper leg musculature during maximal-speed over-ground sprinting. These data from collegiate club hockey athletes corroborate other reports in clinical populations that the coordination between the rectus femoris and biceps femoris is associated with linear over-ground sprinting velocity.

## Key Findings

- Faster collegiate club ice-hockey players had significantly less co-contraction between the rectus femoris and bicep femoris at the knee joint during the swing phase.
- The lower co-contraction in faster sprinters occurred during the end of the swing phase between the rectus femoris and bicep femoris.
- No differences in co-contraction between slower and faster athletes were found between antagonist and agonist pairs at the hip or ankle during the stance phase.

## Background

Muscle activity during maximal speed (upright) sprinting has been thoroughly covered in a systematic review by Howard and colleagues (2018; [1]). Studies indicate peak quadriceps activity during the swing or ‘float’ phase [2, 3]. The quadriceps are also highly active on the ground during the stance phase [4]. Similarly, the hamstrings are involved in the swing and stance phases [4, 5], with peak activity during the late swing phase [3, 6]. Peak gluteus maximus (GM) activation occurs in the stance phase of sprinting [2, 3]. Studies also indicate GM activity during the late swing phase [7]. Peak EMG activity normalized to maximum voluntary isometric contraction (MVIC) occurred during the foot strike (stance phase) and second float phase (late swing phase) in the biceps femoris (BF), gastrocnemius (GN), and GM, but not in the tibialis anterior (TA) and rectus femoris (RF) [8]. Further, EMG values were lowest in the early swing phase for the BF, GN, and GM but highest in the TA and RF. This study is relevant as these athletes sprinted maximally in the upright position [8]. Thus, lower body muscles constantly fluctuate throughout the sprint cycle from active, less active, and highly active. While research demonstrates this activity, less research discusses the harmony of these muscles’ transitions.

Mero (1992; [9]) stated that bi-articular muscles can be antagonistic or synergistic. In the former case, while the antagonist is opposing agonist activity, it provides stiffness and stability to a joint, reducing possible injury. This is critical when an athlete cuts, decelerates, lands, and sprints at high speeds [9]. The simultaneous activation of agonist and antagonist muscles is known as co-contraction, which could hinder performance in certain athletic situations where producing force in minimal time is optimal. For example, increases in RF and BF coactivation increased knee joint stiffness during the late-swing and stance phases in elite Kenyan runners. However, the energy cost of running increased, likely exerting a negative influence on performance at faster running velocities [10]. In recreational runners, higher co-contraction increased energy cost, and slower running increased co-contraction [11]. Also, individuals suffering from musculoskeletal injuries and disorders have higher co-contraction than individuals without musculoskeletal injuries and disorders [12, 13]. Thus, a decent amount of research has demonstrated that co-contraction increases the metabolic cost of the runner. However, scant research exists on the performance outcomes of co-contraction related to sprinting and whether it is associated with sprinting velocity.

Co-contraction of the GM, RF, BF, GN, and TA muscles occurs during the initial contact phase of sprinting [8]. During the swing phase, the RF and BF alternate twice as agonist and antagonist muscles [2, 9]. The BF works as an agonist at the end of the stance phase by extending the hip while the RF antagonistically co-contracts to maintain posture and provide joint stability [2]. Subsequently, the agonist RF flexes the hip and moves the thigh forward in early to mid-swing. This early to mid-swing switch is the ‘first switch’ between the RF and BF. The mid to late-swing switch is the ‘second switch’, where activation transitions from the RF to the BF to extend the hip joint [14]. Also, it is essential during the late swing phase that the RF agonistically extends the knee, and the BF eccentrically, isometrically and antagonistically assists with knee extension [5, 15]. The rapid and frequent change of agonist and antagonist relationships between the RF and BF affects step frequency in elite Japanese track and field athletes with an average 100-m time of 10.46 s [16]. Thus, sprinting requires the rapid and harmonious recruitment of distal and proximal portions of muscles, which consist of activation and relaxation. Many athletes in sports engage in maximal speed sprint bouts, hockey included. Thus, it is a worthwhile endeavor to study possible contributors or inhibitors to better performance. We surmise that the muscular co-contraction activity of agonist and antagonist muscles is a key performance indicator to monitor and understand.

It is well known that ice-hockey athletes engage in off-ice training programs that typically include high-speed sprinting and change-of-direction shuttles, and these periods are considered much-needed breaks from the ice [17, 20]. Moreover, skating speed and 36-m off-ice sprint speed are strongly correlated (*r* = 0.81 *p* < 0.01) [18], and the 40-yard dash and vertical jump are significant predictors of on-ice skating performance specific to speed [19]. Thus, those working to enhance the performance of ice hockey athletes may benefit from this research. In addition, all athletes seeking to improve the function of the nervous system through high-speed sprinting may benefit from examining the findings of this study.

The primary purpose of this study was to compare agonist and antagonist muscle co-contraction during off-ice maximal sprinting between faster and slower club ice hockey players. It is a reasonable position to assume that greater muscular co-contraction indirectly suggests less relaxation and is thus a negative outcome. Thus, we hypothesized that slower athletes would exhibit greater co-contraction than faster athletes. Therefore, a secondary purpose was to explore relative and peak muscle activation between faster and slower athletes.

## Methods

### Experimental Approach to the Problem

#### Subjects

The Florida State University Institutional Review Board (IRB) approved the study. All university club hockey team members were invited to be involved in the study. We studied this population for convenience for this proof-of-concept research among collegiate athletes. Subjects were informed of potential risks and benefits and made aware that participation in the experiment was voluntary. All participants provided written consent before testing. All athletes with an injury in the past six months were excluded. The participants completed a short questionnaire to determine their training history, caffeine intake, and activity familiarity. Only athletes who trained more than one day a week for > two years without a detraining interruption of longer than six months were considered trained (*n* = 12 trained, *n* = 10 untrained). The athletes were instructed to avoid alcohol, caffeine, ergogenic aids, and food intake for three hours before each trial. This study spanned four days and was conducted at the Institute of Sports Sciences and Medicine, Florida State University, Tallahassee, FL.

### Day One Testing

#### Body Mass and Body Composition

Body mass was measured using a force plate (Hawkins Dynamics, ME). Immediately after weighing, body composition was assessed in the seated position using bioelectrical impedance analysis (Omron NBF-306 C).

#### Force Plate Testing

Force-time metrics were assessed via a force plate (Hawkins Dynamics, ME) during a countermovement jump (CMJ), rebound jump, and isometric mid-thigh pull. Participants then engaged in a short dynamic warm-up that consisted of five dynamic stretches of 10 yards each and three submaximal sprints of 70%, 80%, and 90% intensity.

#### Sprint Testing

After a three-minute rest period following the last submaximal sprint, the athletes completed two maximum-effort 20-meter sprints, measured with electronic timing gates (VALD Performance, Australia). The athletes were given a 15-meter acceleration zone and instructed to reach full speed before hitting the first timing gate. The best of two attempts were used in the analyses.

### Day Two Testing

Three days later, the athletes returned individually for EMG analysis. Before sprinting, electrodes, sensors, and foot insoles (Noraxon, AZ) were placed/inserted. The participants executed two 20-yard flying sprints with four minutes between each repetition. We normalized the EMG values to quantify muscle activation using maximum EMG amplitude during the first trial to better assess high-speed EMG activity [7, 21]. Briefly, this method was chosen instead of a traditional (prone/supine) isometric contraction since the traditional isometric contraction is not a good indicator of the activation and high-velocity movements should be normalized to similar, if not identical, movements.

We measured EMG using the Noraxon Software v3.4 device (Noraxon USA Inc., Scottsdale, AZ). Standard sEMG electrodes were positioned with a 2-cm spacing along the longitudinal axis of the muscles on the right leg, based on the anatomical reference points and following the SENIAM guidelines for sensor placement [22]. Muscles were palpated to identify the surface electrode positioning. Each electrode placement site was shaved using a razor and cleaned with alcohol-soaked cotton wool. Next, we secured the electrodes with tape to reduce motion artifacts [15, 23]. Acceptable impedance (noise) was set below ten microvolts (uV). Before data treatment, custom-made digital filtering (Bandpass filter; 20–500 Hz), rectification, and smoothing (Root Mean Square algorithm, 100 ms) was applied to the recorded signal. The reliability of relative muscle activation patterns was calculated using intraclass correlation coefficients (ICC; Version 29, SPSS Statistics, IBM, Armonk, NY, USA). The ICCs were: Swing − 0.03 for GM, 0.80 for TFL, 0.87 for BF, 0.60 for RF, 0.67 for GN, & 0.40 for TA; Stance − 0.83 for GM, 0.79 for TFL, 0.89 for BF, 0.07 for RF, 0.83 for GN, & 0.34 for TA. For context, below 0.50 is considered poor, between 0.50 and 0.75 is considered moderate, between 0.75 and 0.90 is considered good, and above 0.90 is considered excellent [24].

Noraxon myoPRESSURE ™ foot insoles were inserted into the participant’s footwear to determine the swing and stance phases and the application of force by the feet during the stance phase. Participants were instructed to stand on each leg for three seconds to create average pressure plots. Relative EMG activity of the right leg at touchdown and during the early-swing, mid-swing, and late-swing phases were recorded and analyzed. The stance phase was not delineated. When we found significance between groups with the overall swing phase, we parsed apart the swing phases. Specifically, the early swing phase begins when the toes leave the ground and ends one-third through the swing phase. The middle swing begins one-third through the swing phase and ends at approximately two-thirds of the total time to complete the swing phase. The late-swing phase starts about two-thirds of the total time to complete the swing phase and ends at ground contact [4, 5].

Muscular co-contraction, or muscular coactivation, is the simultaneous contraction of agonist and antagonist muscles crossing the same joint [25]. The equation to calculate muscular co-contraction is as follows:

$$\:CCI=\frac{EMGS}{EMGL}*\left\{EMGL*\left(EMGS+EMGL\right)\right\}$$

Where CCI = co-contraction index of two agonist and antagonist muscles.

Where EMGS = percentage activity in the less active muscle relative to the peak EMG for that muscle.

Where EMGL = percentage activity in the more active muscle relative to the peak EMG for that muscle [26].

#### Statistical Analysis

There was no a priori power analysis for this investigation. Athletes were divided into two groups using a median split of maximum-speed sprinting velocity during the 20 m sprint obtained during testing on day one. We compared groups using unpaired, two-tailed t-tests or Mann-Whitney tests when data failed (*p* > 0.05) the Shapiro-Wilk test for normality. A Chi-squared test was also used to compare the proportion of trained adults in each group. Using the EMG data from day two, we compared CCI using mixed effect models with Gait Phase (repeated factor) and Group (Independent factor) separately for the ankle (TA & GN), knee (RF & BF), and hip (GM & TFL) to address our primary hypothesis. Gait phase duration was compared using a mixed effect model with Gait Phase (repeated factor) and Group (Independent factor). Next, muscle activation (expressed as absolute uV & as a percentage of their MVC) was compared using mixed effect models with Gait Phase (repeated factor) and Group (Independent factor) for each of the six muscles examined. Sidak’s multiple comparisons test was employed for all mixed effects models for post hoc analyses. Additionally, Spearman’s correlational analysis was used to relate 20 m maximum-speed sprint velocity with several variables of interest. We present values as mean ± standard deviation (SD) for data normally distributed or median [interquartile range] for data not normally distributed. We analyzed data using Prism (version 10.0 for Windows, GraphPad Software, San Diego, CA, USA). We set α a priori to 0.05.

## Results

We present participant characteristics for the whole cohort and both groups in Table 1. Maximum-speed 20 m sprint velocity was, by design, different between groups. No other factors were significantly different between groups.

**Table 1:** Group characteristics

	All (*n* = 22)	Faster (*n* = 11)	Slower (*n* = 11)	*p*-value (Faster vs. Slower)	
Age, years	21[1]	21[1]	21[2]	0.27	
Body mass, kg	77 ± 9	77 ± 9	77 ± 9	0.87	
Body fat, %	13 ± 3	12 ± 4	15 ± 3	0.06	
Trained in recent ≥ 2 years	14/22	6/11	6/11	> 0.99	
Maximum-speed 20 m sprint velocity, m/s	8.1 ± 0.5	8.5 ± 0.3	7.7 ± 0.3	< 0.001	
Stance time, ms	153 ± 30	153 ± 34	153 ± 27	0.96	
Swing time, ms	305 ± 45	294 ± 35	313 ± 55	0.37	
Early swing time, ms	85[37]	80[33]	98[27]	0.16	
Mid swing time, ms	137[25]	136[25]	145[22]	0.44	
Late swing time, ms	85 ± 20	80 ± 20	88 ± 20	0.39	

We present values as mean ± SD or median [interquartile range] for data that are not normally distributed. The groups were based on the median split of maximum-speed sprinting velocity during the 20 m sprint

### CCI During Over Ground Sprinting

**Fig. 1:**
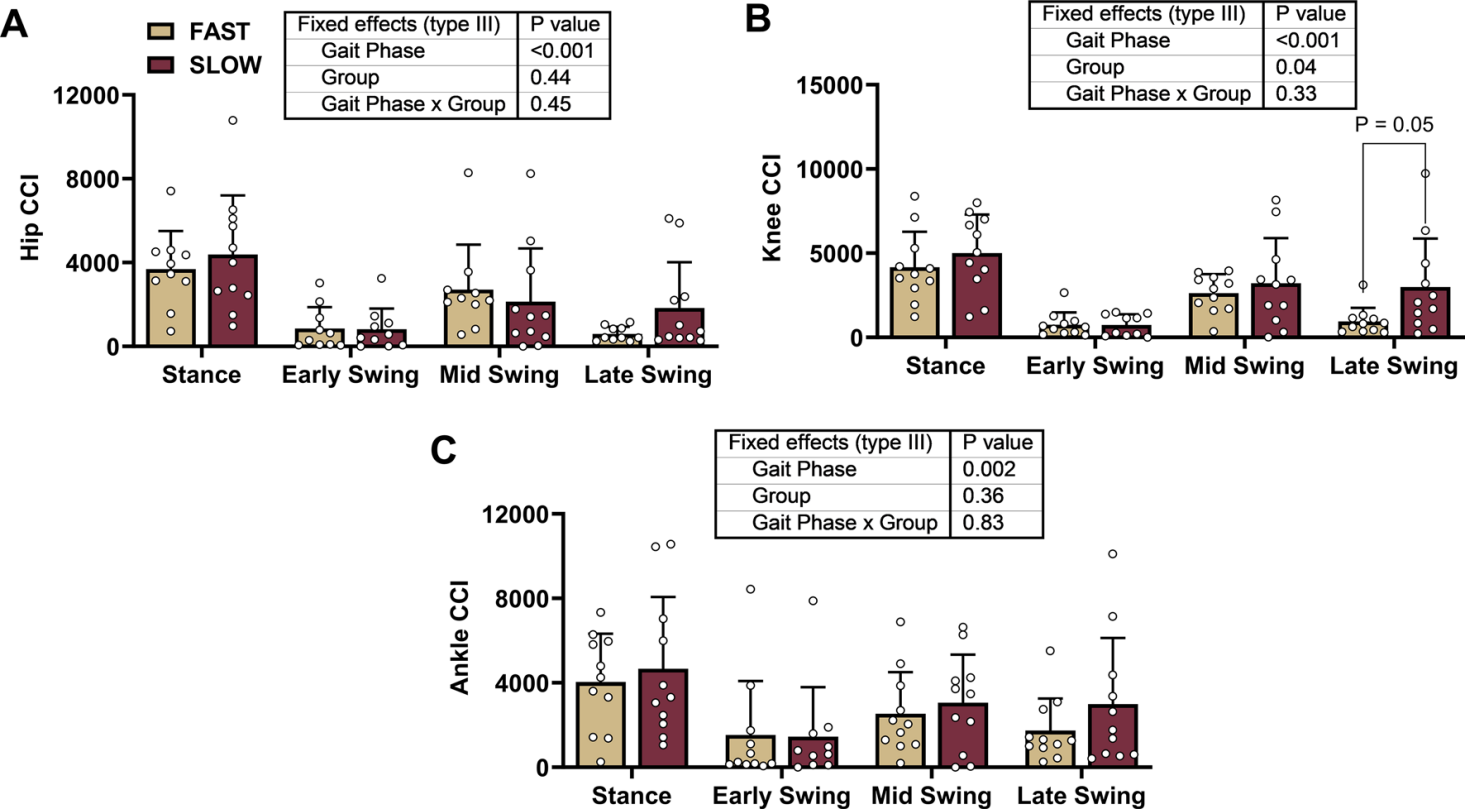
Co-contraction index (CCI) During Over Ground Sprinting. We compared CCI values between groups and gait phases using mixed effects models for the hip **(A)**, knee **(B)**, and ankle **(C)**. We found a significantly lower CCI between the rectus femoris and biceps femoris (i.e., at the knee joint) in faster sprinters relative to the slower sprinters in the swing phase. The gait phase significantly affected the knee and ankle joints but not the gait phase * group. We present individual data with mean ± standard deviation

A significant main effect of the gait phase was found for the knee and ankle, but not hip, joints (Fig. 1). A lower CCI between the RF and BF (i.e., at the knee joint) in faster sprinters than in slower sprinters was found (Fig. 1B). With significance between groups during the swing phase, we explored whether the groups differed during the early, mid, or late swing phases. A lower CCI between the RF and BF in faster sprinters was found during the late swing phase (faster: 948 ± 803 vs. slower: 2997 ± 2867; post hoc *p* = 0.02) but not early or mid-swing phases (post hocs *p* > 0.81) (gait phase: *p* < 0.001, group: *p* = 0.05, gait phase * group: *p* = 0.15). No group differences were found in the hip or ankle joints (Fig. 1A and C).

### Muscle Activation during Over Ground Sprinting

**Fig. 2:**
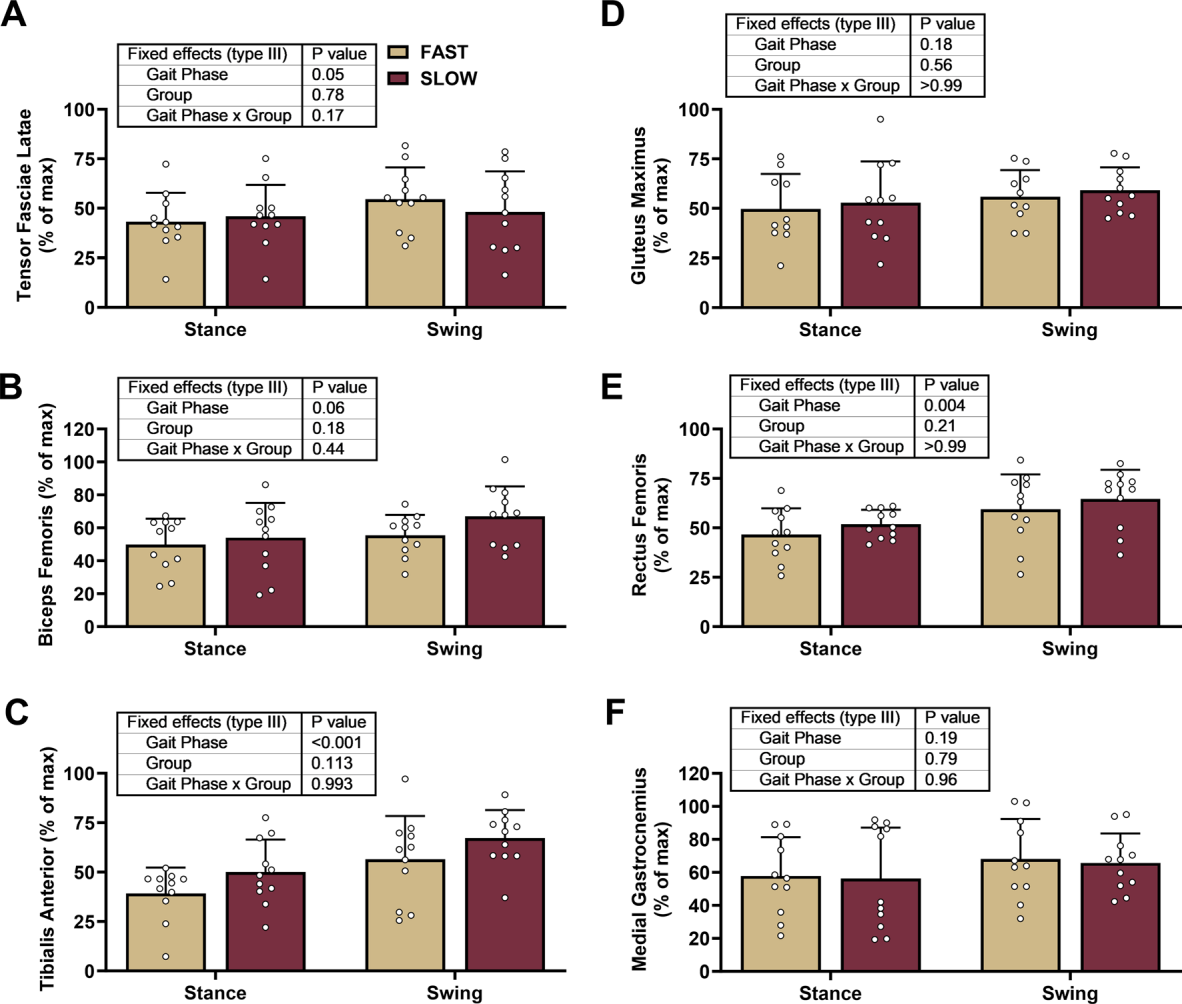
Muscle Activation During Over Ground Sprinting. We compared electromyographic data between groups and gait phases using mixed effects models. The gait phase significantly affected the Tensor Fasiae Latae, Rectus Femoris, and Tibialis Anterior but not the group or gait phase * group. We present individual data with mean ± standard deviation

The gait phase significantly affected TFL, RF, and TA activation but not the group or gait phase * group (Fig. 2A-F). While we did not observe group differences in BF activation (Fig. 2B), we explored whether there were groups during the early, mid, or late swing phases for RF and BF activation based on the significant group differences for knee CCI values in the swing phase (Fig. 1B). Interestingly, there were no differences in muscle activation between groups in the RF or BF during the early, mid, or late swing phases (post hoc *p* ≥ 0.09) (gait phase: *p* ≤ 0.01, group: *p* ≥ 0.24, gait phase * group: *p* ≥ 0.18). Finally, no group differences in GM or GN activation were observed (Fig. 2D and F).

**Fig. 3:**
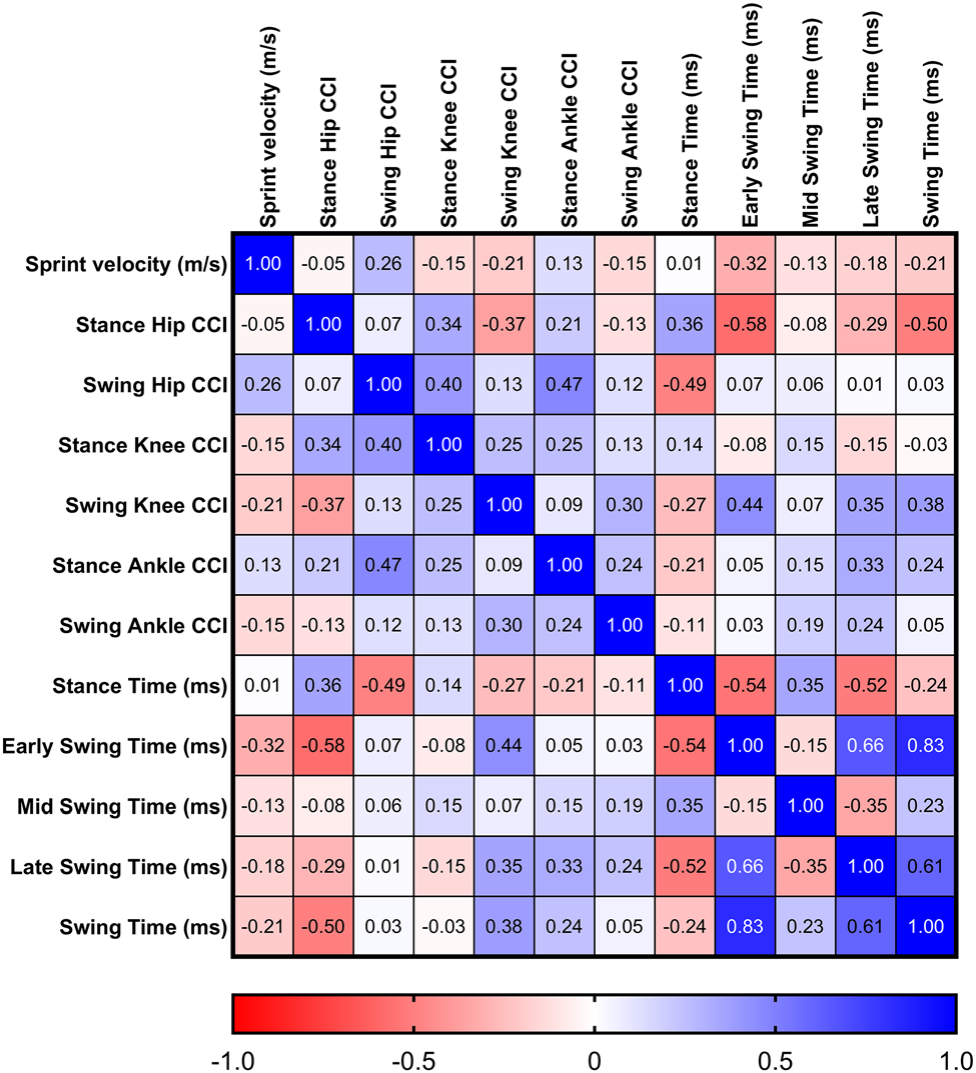
Correlations Between Sprinting Velocity, Co-contraction Index (CCI) values, and Gait Phase Durations. We present Spearman’s rank-order correlation (ρ) from exploratory non-parametric analyses in the boxes (e.g., Stance Hip CCI vs. Sprint velocity ρ=-0.05)

The maximum-speed sprint velocity was not significantly correlated with any CCI values or gait phase durations (*p* > 0.15 for all) (Fig. 3). Stance phase duration was weakly inversely related (*p* = 0.02) to Hip CCI during the swing phase (Fig. 3). Early swing phase duration was moderately inversely related to Hip CCI in the stance phase (*p* < 0.01) and weakly related to Knee CCI in the swing phase (*p* = 0.046) (Fig. 3). Swing phase duration was moderately inversely related to Hip CCI in the stance phase (*p* = 0.02) (Fig. 3). None of the other phase durations were significantly related to CCI variables (*p* > 0.08 for all) (Fig. 3).

## Discussion

The primary purpose of this investigation was to determine if there is more or less muscle co-contraction between faster and slower club hockey players. Also, we wanted to determine the muscle activity of three agonist/antagonist muscle pairs and compare groups. The primary novel finding of this study was that faster collegiate club hockey athletes had lower EMG-assessed CCI values in the knee joint, but not in the hip or ankle joints, during over-ground sprinting. Specifically, the CCI value between the BF and RF CCI was lower in faster sprinters, particularly during the late swing phase. Additionally, we found lower activation of the TA throughout the gait cycle in faster sprinters. These findings indicate that increased relative muscular activation during maximal-speed sprinting was found in slower athletes. Finally, exploratory correlations suggest that time spent in the swing phase, particularly the early swing phase, is moderately inversely related to Hip CCI in the stance phase (*r* = -0.58).

During the swing phase, the primary role of the RF is to swing the thigh forward while its antagonist, the BF, extends the hip in preparation and through ground contact [27]. During the late swing, the RF extends the tibia while the BF contracts to slow the momentum of the tibia as the knee extends immediately before ground contact [5]. The RF activity during the swing phase is considered a hip flexor, not a knee extensor. Thus, abnormally high activation of the leg extensors during the late swing phase is considered atypical [10], which was found to be the case in our study. The RF relative activation percentage was similar between groups during the swing phase. We also found no differences in relative BF and RF activation during the swing phase between groups. In summary, the CCI values for the knee were significant due to higher activation of the BF in slower sprinters. Moreover, increased co-contraction resulted in slower sprinting, which some researchers have encouraged as a possible positive indicator of faster sprinting.

Intentional anticipatory co-contraction or koopretension may reduce muscle slack, resulting in greater stiffness in the series elastic component (tendon) and better performance [28]. While studies support the idea that the contractile elements of the hamstring muscle isometrically contract during the swing phase, there is little evidence to support this theoretical framework during sprinting. To this author’s knowledge, no study has demonstrated that the voluntary act of co-contraction during high-speed sprinting would result in better performance. Moreover, inducing more co-contraction would have the opposite effect based on these data. Van Cutsem and Duchateau (2005 [28]), indicated that pretension before explosive movements reduced the rate of torque development. Thus, intentional muscle co-contraction and its impact on high-speed movement may not be advantageous.

Increased muscle co-contraction may increase muscle and joint stiffness, increasing passive elastic energy return and decreasing muscle slack [29, 30]. While it is generally agreed that increased stiffness improves performance, it is unclear whether this stiffness results from greater muscle co-contraction or mechanical properties of the tendon. Though only one CCI index was statistically significant, of the CCI calculations, 10 of these group means were higher in the slower group than the faster group. Compelling evidence indicates that the hamstrings passively lengthen at the beginning of the swing phase and primarily isometrically contract at the end of the swing phase [28]. Researchers have investigated the progression of passive lengthening to maximal voluntary isometric contractions (MVIC) in these muscles, albeit at slower speeds but at similar joint angles to sprinting. For pilot data, nine participants completed a baseline isometric contraction via a dynamometer at 150 degrees. The participants then completed a trial starting at 120 degrees and were forced into an eccentric contraction to 150 degrees, followed by a maximal isometric contraction (post-stretch isometric contraction). Post-stretch isometric contractions at long muscle lengths resulted in a 20% increase in torque force. In contrast, the muscle activation of the hamstrings considerably decreased compared to maximal isometric performed at 150 degrees [31]. Thus, more significant amounts of muscle activation and co-contraction may be compensatory outcomes related to a lack of tendinous and parallel elastic component elasticity and stiffness. In our study, relative RF activation during late swing was nearly identical between groups, whereas BF relative activation was greater [31].

Kakehata et al. (2023 [16]), indicated that step frequency depends on RF and BF coordination. The authors referenced this coordination in previous articles as ‘switch,’ which is defined as the relaxation period of the BF and RF muscles in the ipsilateral leg. The authors reported significant negative correlations and large effect size with step frequency and running speed (*r* = -0.66 for both; *P* < 0.001 for both) for the onset of muscle activity of the BF known as ‘switch 2’. Switch 2 occurred during the late swing phase before foot contact and had a significant positive correlation with step frequency (*r* = 0.50, *P* = 0.04). The authors concluded that co-contraction of the BF and RF is not advantageous for faster sprinting during the late-swing phase [14]. The authors indicated that greater step frequency resulted in harmonious switching between the BF and RF during the late swing phase [14]. Importantly, step frequency, not step length, is more closely associated with higher velocity in maximal-speed sprinting [32]. This recent work is the only other research to study these two muscles during the late-swing phase. Though different, our study and this study have similar outcomes. In their study, more co-contraction of the RF and BF during the late-swing phase resulted in lower step frequency. Further, more contraction of the RF and BF during the late-swing phase resulted in slower sprinting.

Moore et al. (2014 [11]), indicated a decrease in co-contraction occurs in the flexor and distal muscles at higher velocities due to the shorter duration of tibialis anterior activation. Our study found that faster athletes had lower EMG activity in the TA during sprinting. Relative TFL and RF activation in the mid-swing phase, though not statistically different between slower and faster runners, had a moderate negative correlation to sprint speed. This makes sense as the TFL and RF, both hip flexor muscles, play a crucial role in moving the femur forward and increasing step frequency [32].

### Limitations

This study has clear limitations, including sport specificity, timing of peak muscle activity, limited run-through space, not accounting for fatigue, and equipment/location constraints. First, the reliability for the stance RF EMG activity was poor (ICC = 0.07), but it should be noted the reliability for the stance BF, swing RF, and swing BF was moderate to good (ICC = 0.50–0.89). Second, the cohort consisted of club-level hockey players who may have exhibited sport-specific adaptions on the ice that were not captured in the present data collected during overground sprinting. The timing of peak muscle activation is unknown relative to a specific gait cycle phase. Several studies have indicated that RF activates earlier, followed by the BF with increased running speed [6, 31]. Thus, relative mean values during each swing phase but without precise ‘switch’ moments. The exact moments of muscular activation during each phase are not known. Moreover, the precise moments of peak muscle activation within each phase are unknown, which may be important. As Jonhagen et al. (1996 [8]) noted, no linear relationship exists between the relative or magnitude of EMG activity and exerted muscular force. A stronger muscle may need less activation, and a weak muscle may require more activation, or the opposite can occur. The reasons for these variations are complex and vast. For example, a more relaxed runner may experience less muscle activation and benefit more from the elastic properties of the musculotendon component. This leads to another limitation: the lack of a musculoskeletal model to estimate musculotendon length to limit the inevitable crosstalk of multiple muscles crossing multiple joints. Third, the run-up to reach maximal speed was limited to 15 m. Thus, it is possible that maximum velocity was not reached and kinematics more related to acceleration mechanics could have occurred, which would reduce the usefulness of this study to practitioners seeking to employ training interventions. Fourth, the characterization of training history was not specific and training volume leading up to testing was uncontrolled. Thus, it is possible some others may have been in better condition and/or fatigued, which has been indicated to decrease leg angular velocity, and hip and knee flexion in the swing-phase, which would ultimately affect muscle activation.

## Conclusions

These data indicate that slower club hockey players experience greater co-contraction at the knee joint during over ground maximal-effort sprinting, specifically in the late swing phase. These data provide rationale for human performance practitioners to further consider co-contraction patterns and potentially influence training interventions. We recommend future research studies elite populations to understand whether co-contraction patterns influence performance.

## Data Availability

All data is stored in a repository and can be provided upon request. All EMG data is available in summary form, and each individual Noraxon data printout for each athlete is available. Please email the corresponding authors for access.

## Abbreviations

EMG: Electromyographic
CCI: Co-contraction index
GM: Gluteus maximus
MVIC: Maximum voluntary isometric contraction
GN: Gastrocnemius
TA: Tibialis anterior
RF: Rectus femoris
BF L: Bicep femoris
TFL: Tensor fasciae latae
CMJ: Counter movement jump
EMGS: Level of activity in the less active muscle
EMGL: Level of activity in the more active muscle
